# Pain and Proximal Leg Weakness: An Atypical Presentation of Guillan-Barré Syndrome

**DOI:** 10.7759/cureus.113546

**Published:** 2026-07-28

**Authors:** Julie McDaniel, Aashka Shah, Fang Li, Rizwan Malik

**Affiliations:** 1 Neurology, Carle Bromenn Medical Center, Normal, USA; 2 Surgery, Wellstar Regional Medical Center, Atlanta, USA; 3 Pulmonary and Critical Care Medicine, University of Illinois Urbana-Champaign, Normal, USA

**Keywords:** acute inflammatory polyradiculoneuropathy, areflexia, gullian-barre syndrome, hyponatremia, myalgia, neuropathy, syndrome of inappropriate secretion of antidiuretic hormone (siadh)

## Abstract

Guillain-Barré syndrome (GBS) is an acute, rapidly progressive immune-mediated polyradiculoneuropathy that is a neurologic emergency given the risk of mortality associated with respiratory failure. This report adds to the growing literature on atypical early presentations of GBS.

A 63-year-old man initially presented to the ED with a two-day history of diffuse myalgias and intermittent 'sharp zingers' lasting a few seconds. These started in his hands and quickly spread to his upper and lower extremities. He had recently recovered from an upper respiratory infection. Lab workup was notable for a mild elevation in creatine kinase (CK), and examination revealed no focal deficits. He was discharged home with a diagnosis of post-viral myalgias. The following day, he developed worsening back and leg pain, lower extremity weakness, and increased work of breathing and was readmitted to the hospital. Workup demonstrated areflexia, isolated hip flexor weakness (4/5), and diminished sensation to pin in distal extremities with no upper motor neuron signs.

The patient was clinically diagnosed with GBS and started on intravenous immunoglobulin (IVIG) with improvement. A lumbar puncture (LP) subsequently confirmed GBS with cytoalbuminologic dissociation with CSF of 239 mg/dL (normal range: 15 to 60 mg/dL) and normal WBC count. The patient's hospital stay was complicated by the syndrome of inappropriate antidiuretic hormone secretion (SIADH).

A challenge this case presented was the atypical description of sensory symptoms ('sharp zings' instead of the typical numbness/tingling) and the proximal rather than distal leg weakness. Patients who present with sensory and/or motor deficits require an exam including reflexes and a sensory pin exam to evaluate for GBS given the variable presentation of this condition.

## Introduction

Guillain-Barré syndrome (GBS) is an acute and rapidly progressive immune-mediated polyradiculoneuropathy. It is a neurologic emergency because of the risk of respiratory failure, which occurs in up to 25% of patients. There is significant morbidity, as approximately 20% of patients are unable to walk independently at six months [1,2]. It usually develops in days to a few weeks after a preceding infectious event. Clinical presentation includes ascending, symmetric weakness affecting lower extremities before upper extremities and reduced or absent deep tendon reflexes. It is often accompanied by lower back pain, which can either precede or follow onset of weakness [2,3].

Clinical evaluation may reveal atypical sensory, motor, or autonomic manifestations, as well as hyponatremia, which can delay diagnosis [4,5]. Early recognition is critical, as timely treatment is associated with improved functional outcomes and reduced morbidity. Therefore, awareness of uncommon presentations is essential for clinicians across multiple specialties. This report adds to the growing literature on atypical early presentations of GBS, as it may look like nonspecific pain or post-viral myalgia, and emphasizes the importance of maintaining a broad differential diagnosis when evaluating patients with unexplained neurologic symptoms. 

## Case presentation

A 63-year-old man with a past medical history of chronic atrial fibrillation on Xarelto, cough-variant asthma, longstanding type 2 diabetes mellitus, hypertension, and obstructive sleep apnea presented to the ED with a two-day history of diffuse myalgias and intermittent 'sharp zingers,' described as brief, stabbing pains that migrated between his hands, arms, and feet. He had developed an upper respiratory tract infection 15 days ago for which he had recently completed a course of antibiotics. 

Initial physical examination performed by the ED physician noted intact strength testing; notably, deep tendon reflexes and a detailed sensory exam with pinprick were not performed. Laboratory studies were unremarkable except for a mildly elevated CK level of 243 U/L (reference range: 22-200 U/L). He was diagnosed with post-viral myalgias and was discharged home with follow-up appointments with a neurologist and primary care physician (PCP).

The following day, he presented to his PCP with worsening low back and bilateral lower extremity pain, difficulty walking, numbness/tingling in his arms, and increased work of breathing. His PCP contacted an outpatient neurologist who advised returning to the ED for emergent neurological evaluation. 

**Figure 1:**
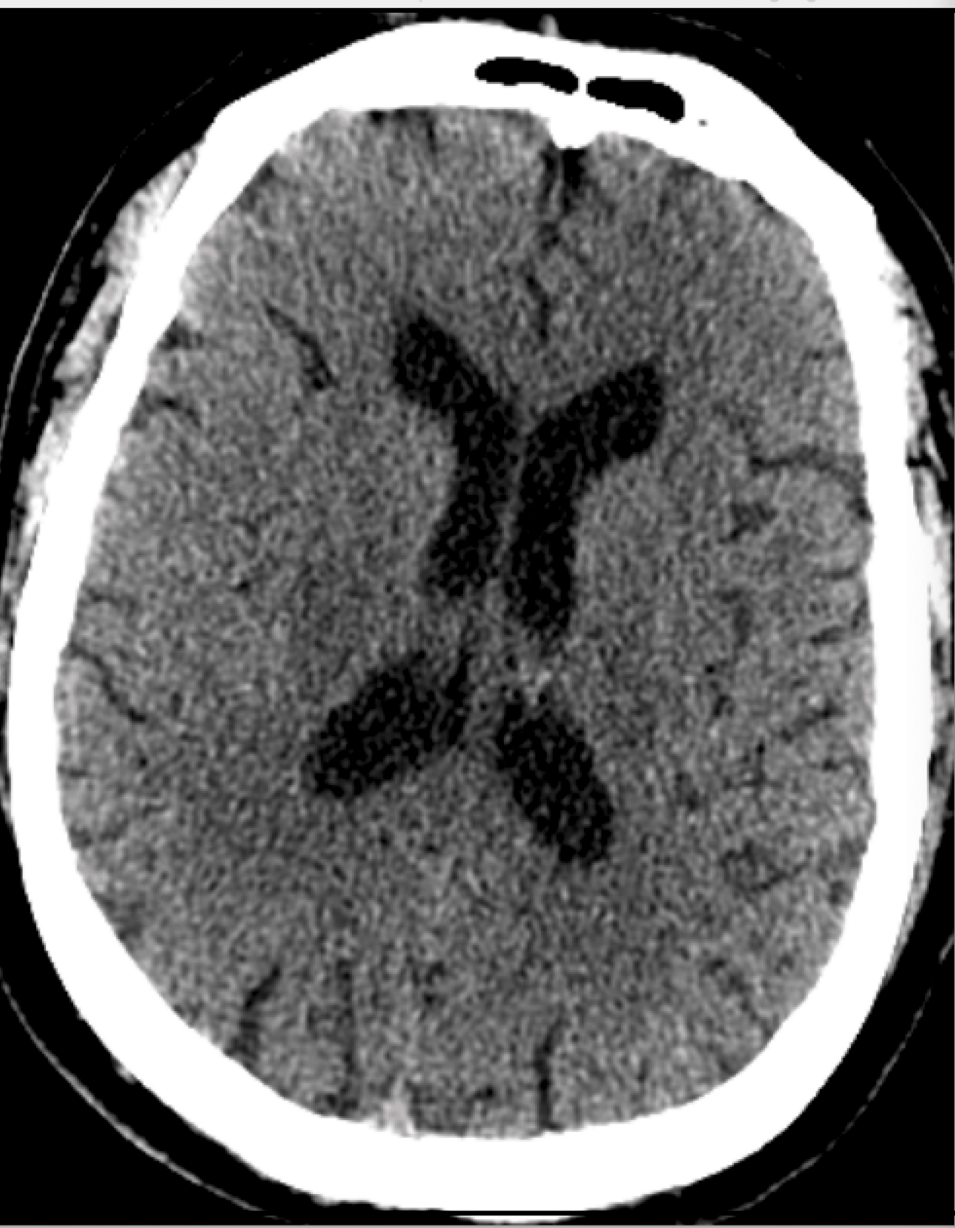
CT of the brain is unremarkable

The ED obtained a head CT, which was unremarkable (Figure 1), and then consulted neurology. The neurologist's exam revealed diffuse areflexia, diminished pinprick sensation extending to the hips in both lower extremities and involving both hands and distal forearms, and symmetric 4/5 hip flexor weakness bilaterally. Distal lower extremity strength was preserved, and no upper motor neuron signs were identified. His gait was unsteady, and cranial nerves 2-12 were intact. The ED laboratory testing revealed an increase in CK to 505 U/L (normal range: 22-200 U/L), erythrocyte sedimentation rate (ESR) of 39 mm/hr (normal range: 0-30 mm/hr), and C-reactive protein (CRP) of 8.8 mg/L (normal range: 0-9.9 mg/L) (Table 1).

**Table 1:** Laboratory values CK: Creatine kinase, ESR: Erythrocyte sedimentation rate, CRP: C-reactive protein

Parameters	Patient values	Normal range
Serum CK (day 1)	505 U/L	20-200 U/L
Serum ESR (day 1)	39 mm/hr	20-40 mm/hr
CRP (day 1)	8.8 mg/L	0-9.9 mg/L
Serum sodium (day 1)	130 mEq/L	135-145 mEq/L
CSF protein (day 2)	239 mg/dL	15-60 mg/dL
Serum sodium (day 3)	123 mEq/L	135-145 mEq/L
Serum osmolality (day 3)	264 mOsm/kg	275-300 mOsm/kg

Based on the ascending, progressive sensory loss with areflexia, the clinical impression was GBS. The preceding upper respiratory infection also matched GBS presentation. The preservation of distal strength with weak hip flexors was atypical, but the neurologist reviewed case reports noting initially isolated hip flexor weakness being confirmed to be GBS [5]. The prior diagnosis of post-viral myalgias would not explain the evolving sensory loss, weakness, or areflexia. Inflammatory myositis was considered, but the prominent sensory symptoms and areflexia were inconsistent with this diagnosis. Polymyalgia rheumatica (PMR) was considered, but he lacked classic morning stiffness and had a normal CRP (99% of PMR cases have elevated CRP); therefore, GBS was a more fitting diagnosis. Immediate treatment with intravenous immunoglobulin (IVIG) vs. plasma exchange (PLEX) was offered, but the patient opted to wait as he considered the treatment options and awaited further test results. Xarelto was held pending lumbar puncture (LP). 

**Figure 2:**
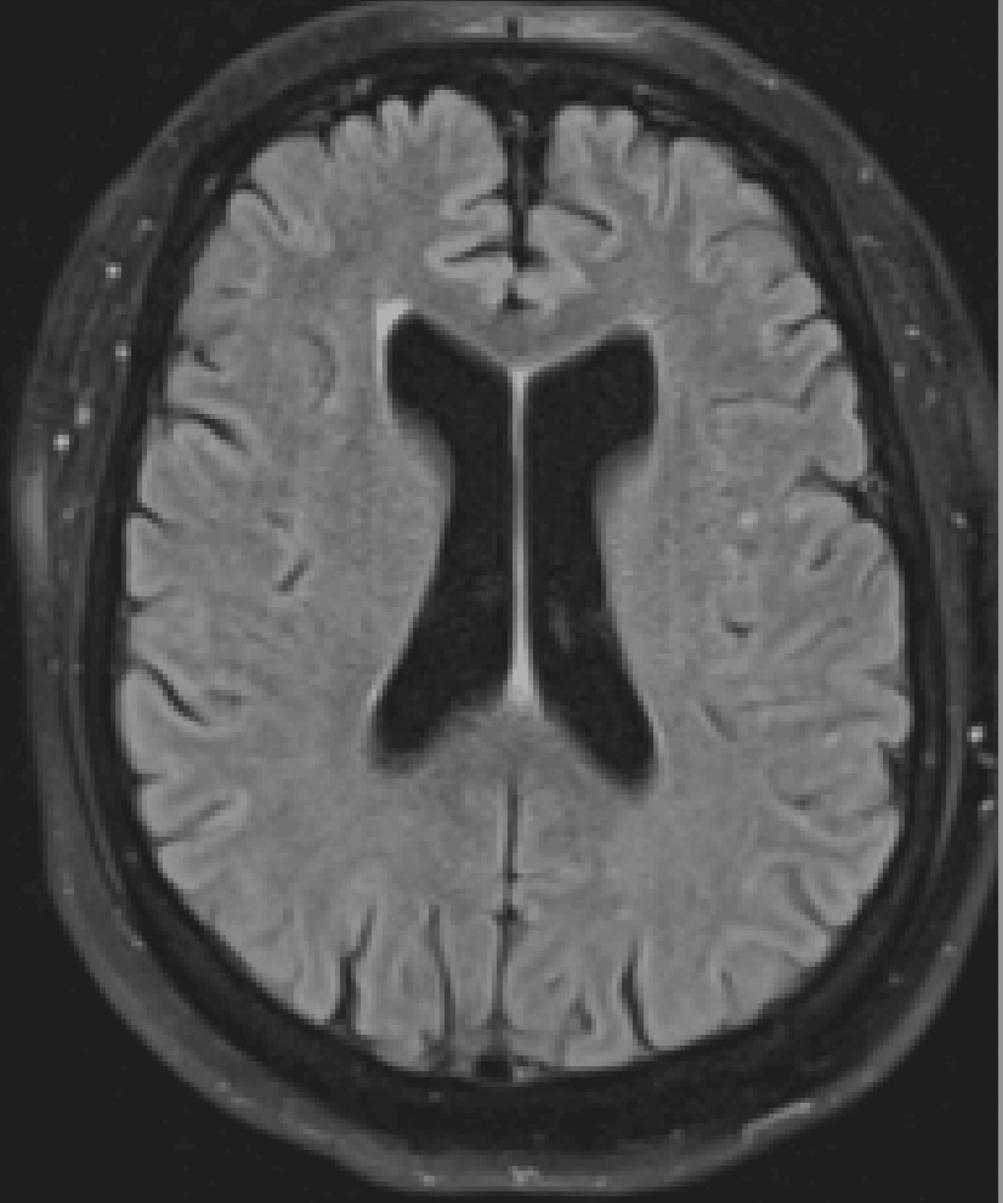
MRI of the brain is unremarkable

**Figure 3:**
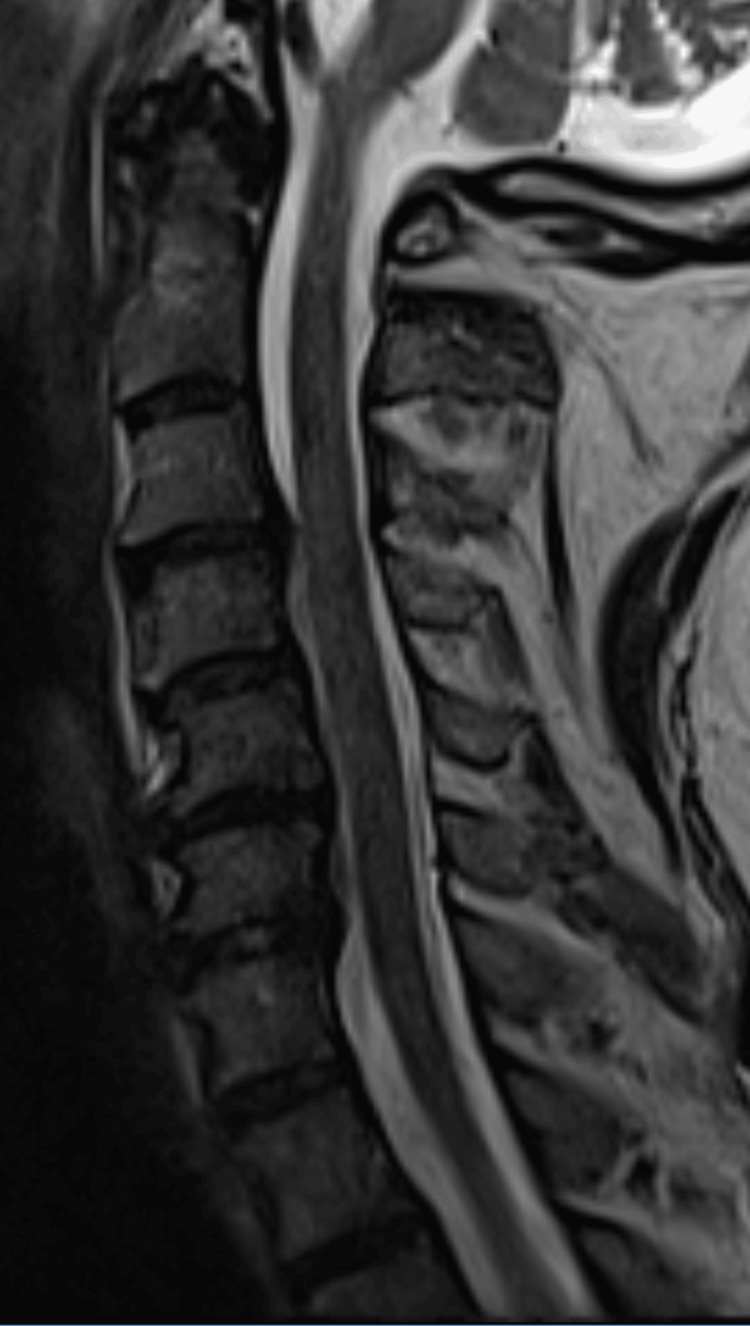
MRI of the cervical spine is unremarkable

**Figure 4:**
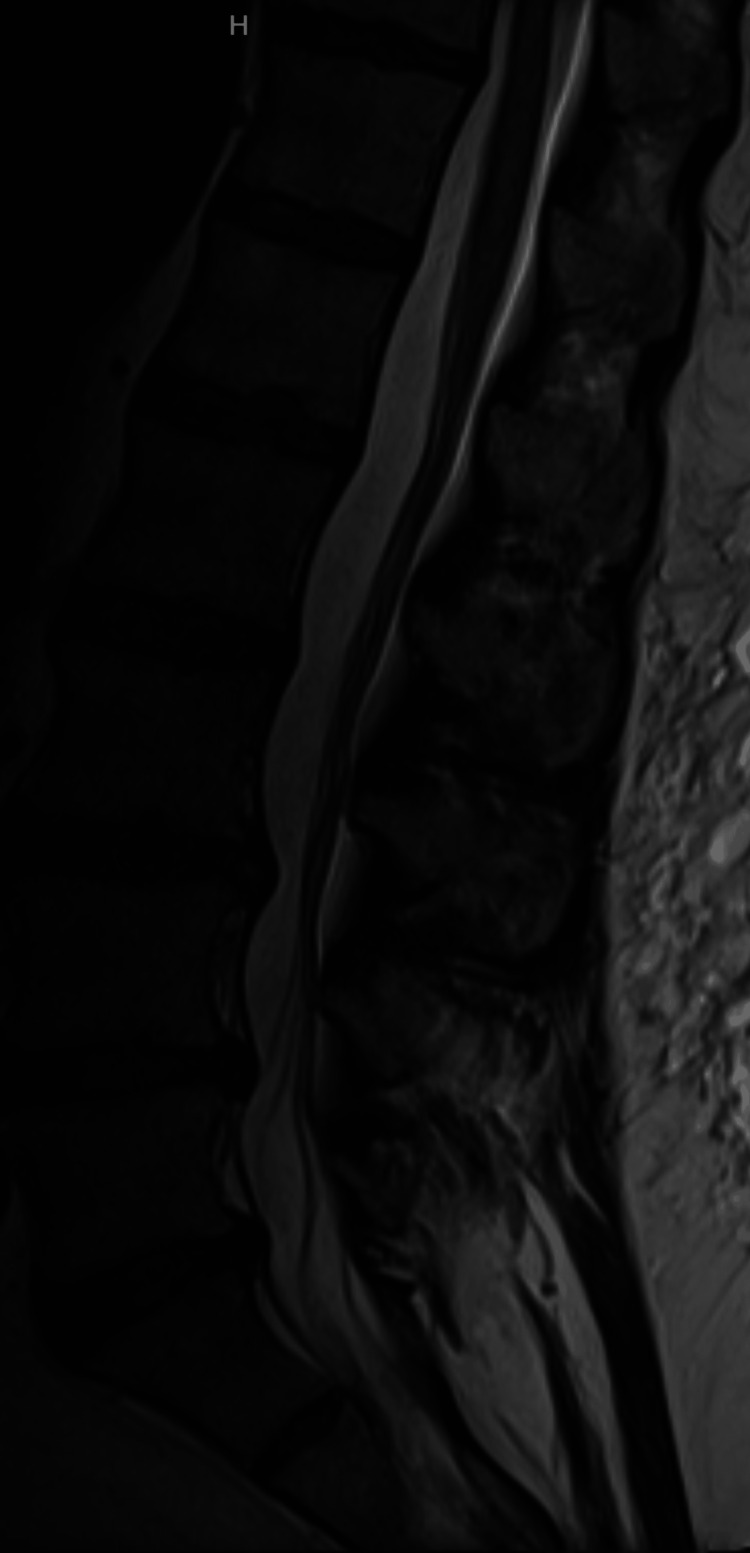
MRI of the lumbar spine is unremarkable

Although further testing was not required to make the clinical diagnosis of GBS, it was performed to rule out alternative causes of neurologic symptoms. On the second day of hospitalization, the patient had an MRI of the brain (Figure 2), cervical spine (Figure 3), and lumbar spine (Figure 4) performed to rule out GBS mimics such as central nervous system lesions or radiculopathies. The MRIs were normal and excluded those diagnoses. An LP was performed, and CSF studies were notable for cytoalbuminologic dissociation with a protein level of 239 mg/dL (normal range: 15 to 60 mg/dL) (Table 1) and a normal WBC when adjusted for RBC count, further supporting the GBS diagnosis. An inpatient electromyogram (EMG) was not available. The patient subsequently agreed to IVIG therapy, which was initiated the same day. He was treated with IVIG at 400 mg/kg/day for five consecutive days with acetaminophen and diphenhydramine premedication.

The patient was initially admitted to the step-down unit with monitoring of forced vital capacity (FVC) and negative inspiratory force (NIF) every four hours. Within the first 24 hours of his hospitalization, his NIF value dropped from -40 cm H₂O to -20 cm H₂O (normal range: -80 cm H₂O to -100 cm H₂O), and FVC dropped from 2.5 L to 1.8 L (normal value: 4.75 L to 5.5 L), prompting transfer to the intensive care unit due to concern of impending respiratory failure. His NIF the following day was -60 cmH₂O, so it was suspected that the -20 cmH₂O reading was an inaccurate reading due to differences in technique. He was evaluated by the intensivist before he was deemed stable enough to be monitored by the hospitalist service with neurology onboard. His severe lower extremity and low back pain required treatment with alternating morphine and hydrocodone in addition to gabapentin 300 mg three times daily. 

In addition, the patient had presented with sodium levels of 130 mEq/dL at the time of admission to the ED prior to any administration of IVIG, which was attributed to the syndrome of inappropriate antidiuretic hormone secretion (SIADH) associated with GBS. The lowest level of sodium was 123 mEq/L (normal range: 135-145 mEq/L) (Table 1) three days after his presentation, following two doses of IVIG. He had low serum osmolality at 264 mOsm/kg (normal range 275-300 mOsm/kg), which suggests IVIG may have contributed to true hyponatremia as the high sugar content draws water from cells into the bloodstream. His sodium values normalized with a fluid restriction. The patient had resolution of his shortness of breath and significant improvement in his sensory and strength symptoms over the next five days. After one week of hospitalization, he was discharged to acute inpatient rehab for two weeks and subsequently discharged home. 

He was seen by outpatient neurology three months after GBS, and his neurological examination revealed normal gait without use of an assistive device, minimal Achilles reflex, intact patellar reflex, no definite sensory loss on exam, and isolated toe extensor weakness. He was experiencing burning pain in his feet and increased sensitivity in his fingertips. Outpatient EMG of the upper and lower extremities was planned but was ultimately performed only on his upper extremities. Notably, his symptoms were most prominent in the lower extremities, so this decreased the sensitivity of the EMG, which showed median and ulnar neuropathies. 

## Discussion

At initial presentation, the patient's symptoms appeared most consistent with post-viral myalgias given his recent upper respiratory infection, diffuse myalgias, and lack of focal neurologic deficits appreciated. However, his initial exam did not include reflexes or a detailed sensory pin exam. Although the patient's mildly elevated CK initially appeared suggestive of a primary myopathic process, transient CK elevation has been reported in approximately 16% of patients with GBS, particularly in axonal variants, and has a less favorable prognosis [6]. His diffuse muscle aches also pointed the ED physician in this direction, so it is worth recognizing that pain is a common early manifestation of GBS; the Dutch GBS Study Group report pain in approximately 66% of patients during the first three weeks of illness [7]. Guillain-Barré syndrome may present with a variety of clinical manifestations initially, which underscores the importance of keeping this condition in the differential diagnosis beyond classic cases [8]. 

An additional diagnostic challenge was the atypical nature of the patient's sensory symptoms. Rather than the classic distal numbness or tingling, he described intermittent migrating 'zingers,' 'zaps,' and 'needle-like' sensations affecting different limbs. This case illustrates the heterogeneity of early sensory manifestations in GBS and emphasizes the importance of a detailed neurologic examination, as pinprick testing demonstrated significant, objective sensory deficits.

Although CSF analysis is recommended during the diagnostic evaluation, the World Health Organization recognizes GBS as a clinical diagnosis, and cytoalbuminologic dissociation is not required for diagnosis [9,10]. Furthermore, up to 10% of patients have normal CSF protein concentrations early in the disease course, with protein elevation not developing until one to two weeks after symptom onset [11].

The patient's hyponatremia was more severe than is typically observed in GBS; however, this is not an isolated finding, as SIADH has been reported in up to 50% of patients with GBS, with varying degrees of severity [12]. Intravenous immunoglobulin therapy can exacerbate or cause hyponatremia as well via two different mechanisms. It has stabilizers with high sugar content that cause water to shift out of cells into the bloodstream. This is associated with low serum osmolality and is considered true hyponatremia. Intravenous immunoglobulin has a large infusion volume with high protein load that can displace water, causing a falsely low lab reading called pseudohyponatremia associated with normal serum osmolality [13]. 

The patient questioned whether the assessment of deep tendon reflexes and detailed sensory exam with a pin during his initial ED visit might have led to an earlier diagnosis. Fortunately, he saw his PCP the next day, which led him to be evaluated that night by the inpatient neurologist. He was grateful that he was diagnosed and treated in a timely manner with significant clinical improvement. He hopes his case could help other GBS patients with atypical presentations. 

## Conclusions

Guillain-Barré syndrome can present with a wide spectrum of clinical manifestations, making early diagnosis challenging. Clinicians should maintain a high index of suspicion for GBS in patients presenting with unexplained sensory and/or motor deficits. Careful neurological examination, including assessment of deep tendon reflexes and pinprick sensation, provides important early diagnostic clues. Prompt recognition facilitates timely initiation of immunotherapy and supportive care. Early diagnosis and treatment are associated with improved outcomes, including preservation of independent mobility and respiratory function.
